# Tyramine Derivatives Catalyze the Aldol Dimerization of Butyraldehyde in the Presence of *Escherichia coli*

**DOI:** 10.1002/cbic.202200238

**Published:** 2022-07-14

**Authors:** Jonathan A. Dennis, Joanna C. Sadler, Stephen Wallace

**Affiliations:** ^1^ Institute of Quantitative Biology Biochemistry and Biotechnology School of Biological Sciences University of Edinburgh King's Buildings, Alexander Crum Brown Road Edinburgh EH9 3FF UK; ^2^ EaStCHEM School of Chemistry University of Edinburgh, King's Buildings David Brewster Road Edinburgh EH9 3FJ

**Keywords:** biocompatible chemistry, bioorganic chemistry, organocatalysis, whole cells

## Abstract

Biogenic amine organocatalysts have transformed the field of synthetic organic chemistry. Yet despite their use in synthesis and to label biomolecules *in vitro*, amine organocatalysis *in vivo* has received comparatively little attention – despite the potential of such reactions to be interfaced with living cells and to modify cellular metabolites. Herein we report that biogenic amines derived from L‐tyrosine catalyze the self‐aldol condensation of butanal to 2‐ethylhexenal – a key intermediate in the production of the bulk chemical 2‐ethylhexanol – in the presence of living *Escherichia coli* and outperform many amine organocatalysts currently used in synthetic organic chemistry. Furthermore, we demonstrate that cell lysate from *E. coli* and the prolific amine overproducer *Corynebacterium glutamicum* ATCC 13032 catalyze this reaction *in vitro*, demonstrating the potential for microbial metabolism to be used as a source of organocatalysts for biocompatible reactions in cells.

## Graphical abstract

**Bacterial amine organocatalysis**: Biogenic amines derived from L‐tyrosine were found to catalyze the biocompatible self‐aldol dimerization of butanal in the presence of *Escherichia coli*. Cell lysates from both *E. coli* and *Corynebacterium glutamicum* were also found to catalyze this reaction *in vitro*. Under biocompatible conditions, the product enal was reduced to 2‐ethylhexanal via endogenous reductases.

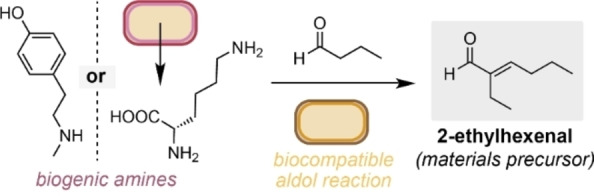

## Introduction

**Figure 1:**
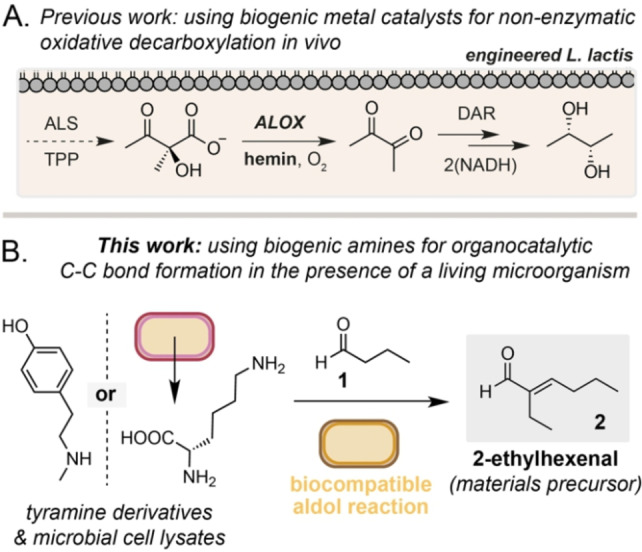
Biocompatible reactions in living microorganisms. A) Hemin catalyzed ALOX chemistry during engineered 2,3‐butanediol biosynthesis in *L. lactis*. B) Biogenic tyramine and cell lysate organocatalysts for the biocompatible self‐aldol reaction of butyraldehyde in the presence of *E. coli*.

The emerging field of biocompatible chemistry aims to combine non‐enzymatic reactions with native and engineered metabolic pathways in living microorganisms.^[1]^ This enables existing catalytic reactions developed in organic chemistry to be applied *in vivo* to enable the bio‐production of non‐natural compounds of industrial importance that cannot be accessed through biosynthesis alone and would otherwise be produced via semi‐synthesis or total synthesis from fossil fuels. Notable achievements in this field in recent years have included the use of photoactivated InP nanoparticles to regenerate NADPH from NADP^+^ in the cytosol of *Saccharomyces cerevisiae*.^[2]^ Irradiation of cells and photoexcitation of the membrane‐bound photocatalyst resulted in a 35‐fold increase in shikimic acid production from dehydroshikimic acid in a NADPH‐deficient ▵*zwf1* knockout strain. Intracellular biocompatible reactions have also been employed to bridge two metabolic pathways in an engineered strain of *Lactococcus lactis*. Here, Fe(III) in hemin was used to catalyze the non‐enzymatic oxidative decarboxylation (ALOX) of α‐acetolactate to diacetyl. This was then intercepted by a heterologous reductase to produce (*S,S*)‐2,3‐butanediol and regenerate NAD^+^ in the cell (Figure 1A).^[3]^ More recently, transition metal catalysts localized in biocompatible TPGS micelles have been interfaced with styrene production from D‐glucose in engineered *E. coli*. Maaskant *et al*. generated styrene from L‐phenylalanine in an engineered *E. coli* C43PF strain (*E. coli* C43(DE3) transformed with a pTrcHis plasmid encoding for *AtPAL2* and *ScFDC1* genes) and used a solid‐supported Zhan II Ru catalyst to produce stilbene *in vivo*.^[4]^ Similarly, Wallace *et al*. expressed the same heterologous genes in *E. coli* NST74 to generate styrene from D‐glucose and interfaced this strain with an Fe(III) phthalocyanine catalyst to form phenylcyclopropanes *in vivo*.^[5]^ In this latter example, the Fe‐catalyzed reaction was accelerated in growth media and in the presence of cells relative to when the reaction was performed in water. This intriguing observation suggests that many reactions developed for use in aqueous solvent in the field of green chemistry may not only be applicable but also improved when conducted in the presence of a microorganism. For this reason, the examination of aqueous‐compatible organic reactions in the presence of living bacteria is an important first step in the development of new biocompatible reactions. To this end, Domaille *et al*. recently demonstrated that α‐ and β‐amino acids mediate an organocatalytic aldol reaction in the presence of *E. coli* cells and that this reaction can be coupled to the oxidation of alcohol substrates to aldehydes by *Gluconobacter oxydans* DSM 2003 and *Komagataella pastoris* ATCC 28485.^[6]^ Inspired by this study we decided to focus on expanding the scope of this screen to encompass organocatalysts used in organic chemistry, biogenic amines that are hypothesized to act as organocatalysts in Nature, and to assess whether native amines generated by living microbes could be used as a source of organocatalysts. We focused our studies on the self‐aldol condensation of butyraldehyde, **1**, in the presence of living *E. coli* as **1** is a known metabolite that can be generated via engineered metabolism and the aldol‐product 2‐ethylhexenal (2‐EH, **2**) is a precursor to 2‐ethylhexanol (2‐EHAO), an important industrial chemical. Currently, **2** is manufactured from propylene generated from the cracking of fossil fuel‐derived hydrocarbons during oil refining and then elongated via Rh‐catalyzed hydroformylation using pressurized CO and H_2_. Furthermore, *E. coli* possesses a series of putative ene‐reductases in its genome with homology to known enoate reductases from *G. oxydans* (44 % identity, Table S5), meaning that 2‐EH could potentially be further processed to 2‐ethylhexanal (2‐EHA, **3**) via endogenous enzymes when generated by biocompatible organocatalysis *in vivo*. Herein we report that *para*‐tyramine and *N*‐methylated congeners including hordenine catalyze the self‐aldol reaction of aliphatic aldehydes in the presence of living *E. coli*. The reaction is biocompatible, affords enals and saturated aldehyde products and can be catalyzed using endogenous amine organocatalysts produced from cultures of *E. coli* and *C. glutamicum* (Figure 1B).

## Results and Discussion

**Figure 2:**
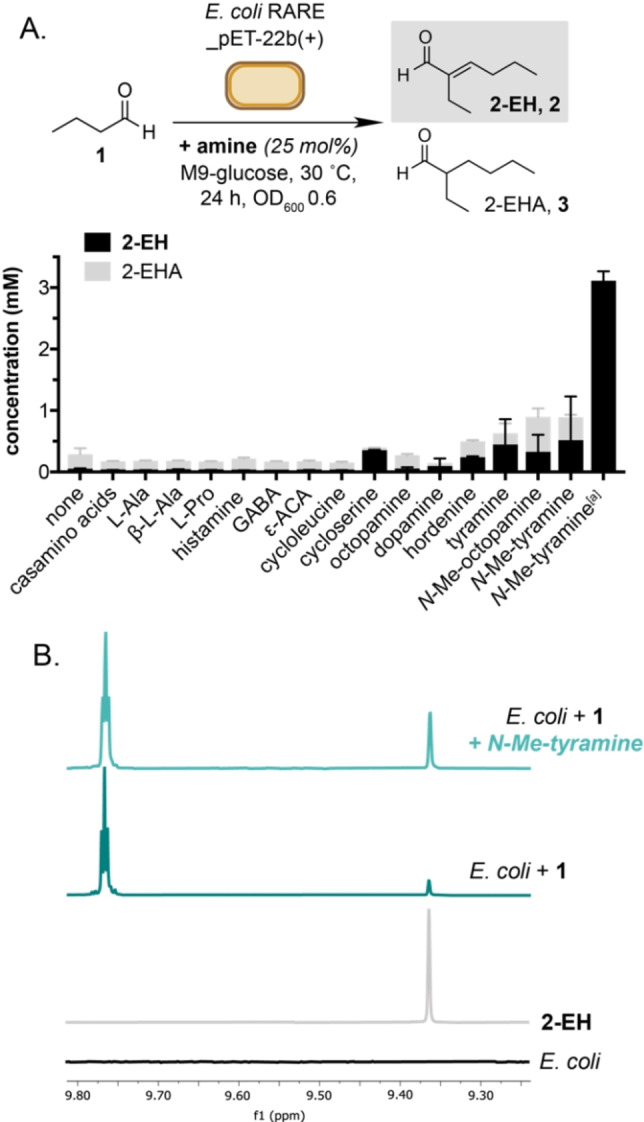
Catalyst screen in the presence of *E. coli*. A) Catalyst screening in cell culture. B) ^1^H NMR analysis of cultures containing amine catalysts. Reactions were conducted with butanal (25 mM), amine (6.25 mM) in sealed Hungate tubes, in the presence of ampicillin (100 mg L^–1^) and under an atmosphere of air. *E. coli* RARE cells transformed with an empty pET‐22b(+) expression plasmid were used (OD_600_ 0.5‐0.6) and reactions were incubated at 30 °C with shaking at 220 rpm for 24 h. Product concentrations in culture extracts were determined by ^1^H NMR spectroscopy relative to an internal standard of 1,3,5‐trimethyoxybenzene. All data are shown as an average of three independent experiments to one standard deviation. [a] 25 mM **1**.

**Figure 3:**
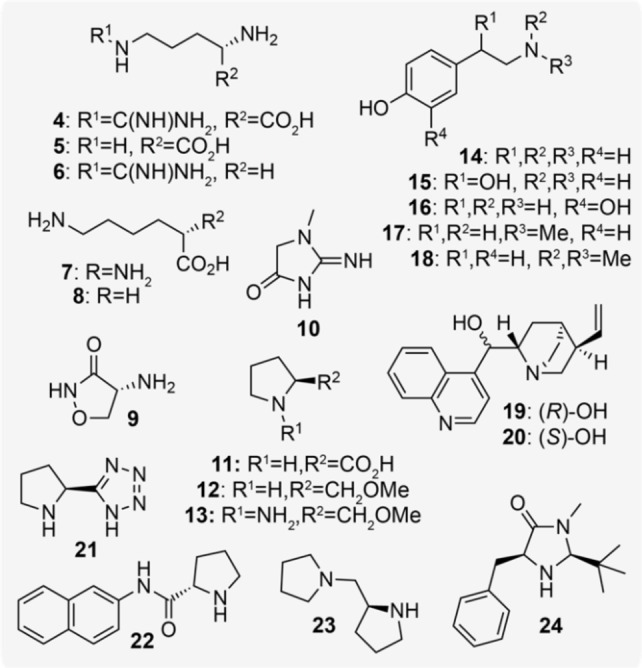
Structures of amine organocatalysts.

We began our study by screening various organocatalysts *in vivo* with a focus on amines that are present in cells or can be accessed via engineered metabolism. *E. coli* “RARE” (*E. coli* MG1655(K‐12)▵*dkgB▵yeaE▵dkgA▵yqhD▵yjgB*▵*yahK*▵*yqh*) was selected for this screen as the strain contains seven gene knock‐outs that enable the accumulation of aldehydes *in vivo*.^[7]^ Butyraldehyde (25 mM) and amines (25 mol%) were added to cultures of *E. coli* MG1655 RARE at mid‐log phase growth (OD_600_=0.6, pH 7) and incubated at 30 °C for 24 h before the product was extracted and analyzed by GC‐FID or ^1^H NMR. Negligible product formation was observed in the absence of any catalyst and 90 % of the product formed in this negative control was present as 2‐EHA, indicating that *E. coli* reduces the C=C bond generated from the self‐aldol condensation of **1**. This aligns with our recent observation that unmodified *E. coli* BL21(DE3) cells can reduce the C=C bond of keto‐acrylates via an unknown mechanism.^[8]^ Addition of the α‐amino acids L‐Gly, L‐Ala, L‐Arg, L‐Asp, L–Cys, L‐Glu, L‐His, L‐Leu, L‐Lys, L‐Met, L‐Phe, L‐Pro, *trans*‐4‐hydroxy‐L‐proline, L‐pyroglutamate, L‐Ser, L‐Thr, L‐Trp, L‐Tyr, L‐Val did not increase product formation (Table S1). This was particularly surprising for L‐Pro and L‐Ala, as these have been used to catalyze the aldol reaction under aqueous conditions *in vitro* and in the presence of *E. coli* at higher substrate concentration.^[6]^ Addition of D‐Pro also resulted in <2 % yield, indicating that the reduced activity of L‐Pro was not due to metabolic utilization of the catalyst in the cell during log‐phase growth. The β‐amino acid β‐L‐Ala increased the yield to 21 %, consistent with reports showing the dimerization of butanal in suspended whole‐cells at 500 mM catalyst loading. No alternative side‐products were detected in culture extracts and butanal was returned in >80 % yield in all cases. We therefore moved on to screen other biogenic amines known to be present in *E. coli* (Figure 3, Table 1 and S1). The decarboxylated α‐amino acid precursors histamine, γ‐aminobutyric acid (GABA) and ϵ‐aminocaproic acid were also inactive, in addition to the amino acid catabolites creatinine **10** and sarcosine, and the non‐proteogenic amino acids norvaline, ornithine and terleucine (Table S1). Other amine‐containing metabolites such as thiamine, uracil and guanidine also had no effect on the yield of **2**/**3**. The cyclic amino acid analogues D‐cycloserine and cycloleucine were both inactive, and product selectivity was reversed using cycloserine **9**. This is likely due to the antibiotic properties of D‐cycloserine arresting cell growth prior to the reduction of **2**. Product yield was increased, however, upon addition of biogenic 1,4‐disubstituted phenethylamines derived from L‐Tyr (Figure 2A). For example, hordenine increased the yield of **2** to 38 %, whereas octopamine and tyramine increased the yield of **2** to 19 % and 25 %, respectively (Figure 2B and Table S1). Reactivity was reduced using the isomeric hydroxyl‐containing analogues *m*‐octopamine and dopamine. However, *N*‐methylation of octopamine and tyramine increased the overall product yield 8‐fold (Table 1 and S1). This led us to hypothesize that increasing the basicity of the terminal nitrogen atom via alkylation increases product formation by promoting the enolization of **1**. This was confirmed by buffering the reaction to pH 9 and observing an increase in yield to 25 % **2** for *N*‐methyltyramine **17** (Table 1, entry 3). *E. coli* is known to grow in alkaline media and cells were found to be viable at this pH (Figure S11). Interestingly, the reactivity of the basic amino acids L‐Lys and L‐Arg were also increased at pH 9, and this could be improved via *N*‐methylation at the α‐position (Table S1). *N*‐methylation of L‐Lys at the ϵ‐position also increased the product yield from 22 % to 27 %. In line with Domaille *et al*., we found that increasing the concentration of the amine catalysts to 25 mM had a further positive effect on the yield of **2**/**3**. For L‐Lys and L‐Arg this increased the yield of 2‐EH to 26 % and 33 %, respectively (Table 1, entries 15–17). Similarly, this increased the yield to 35 % for *N*‐methyltyramine and 38 % for hordenine (Table 1, entries 5 and 8).

**Table 1:** Catalyst screen in the presence of *E. coli* RARE.^[a]^

Entry	Amine [mol %, pH]	2‐EH [%]	2‐EHA [%]
1	none [n/a,7]	<1	6
2	none [n/a,9]	13	2
3	*N*‐Me‐tyramine [25,9], **17**	25	2
4	*N*‐Me‐tyramine [100,9], **17**	35	<1
5	*N*‐Me‐tyramine [100,7], **17**	20	<1
6	*N*‐Me‐tyramine [100,9],^[b]^ **17**	19	1
7	*N*‐Me‐octopamine [100,9]	27	<1
8	hordenine [100,9], **18**	38	n.d.
9	tyramine [100,9], **14**	19	<1
10	octopamine [100,9], **15**	25	2
11	*m*‐octopamine [100,9]	24	<1
12	*N*‐methylphenethylamine [100,9]	27	n.d.
13	*m*‐*N*‐Me‐tyramine [100,9], *meta*‐**17**	18	n.d.
14	*o*‐*N*‐Me‐tyramine [100,9], *ortho*‐**17**	22	n.d.
15	L‐Pro [100,9], **11**	16	7
16	L‐Lys [25,7], **7**	2	6
17	L‐Lys [25,9], **7**	22	<1
18	L‐Lys [100,9], **7**	26	<1
19	L‐Arg [25,7], **4**	2	8
20	L‐Arg [100,9], **4**	33	<1
21	agmatine [100,9], **6**	27	6
22	creatinine [100,9] **10**	14	3
23	Me‐Lys‐OH [25,9]	27	<1
24	Me‐Lys‐OH [100,9]	35	<1
25	(*R*)‐(‐)‐cinchonidine [25,9], **19**	17	<1
26	(*R*)‐(‐)‐cinchonidine [25,9],^[c]^ **19**	21	<1
27	(*S*)‐(+)‐2‐methoxymethyl‐pyrrolidine [100,9], **12**	24	n.d
28	L‐(pyrrolidinylmethyl)pyrrolidine [100,9], **23**	30	<1
29	(*S*)‐1‐amino‐2‐(methoxymethyl) pyrrolidine [100,9], **13**	14	<1
30	L‐Pro‐β‐naphthylamide [100,9], **22**	15	n.d.
31	L‐Pro‐tetrazole [100,9], **21**	17	7
32	MacMillan 2^nd^ Gen [25,9], **24**	15	2
33	(NH_4_)_5_[Fe(C_6_H_4_O_7_)_2_] [10,7]	2	7
34	Zn(L‐Lys)_2_ [100,9]	25	<1
35	Zn(L‐Arg)_2_ [100,9]	16	<1
36	Zn(L‐Pro)_2_ [100,9]	18	<1

[a] Reactions were performed as described in Figure 2. [b] Reaction was performed at room temperature. [c] 10 % vol/vol DMSO was added. All data are shown as an average of three independent experiments. n.d.=not determined.

**Figure 4:**
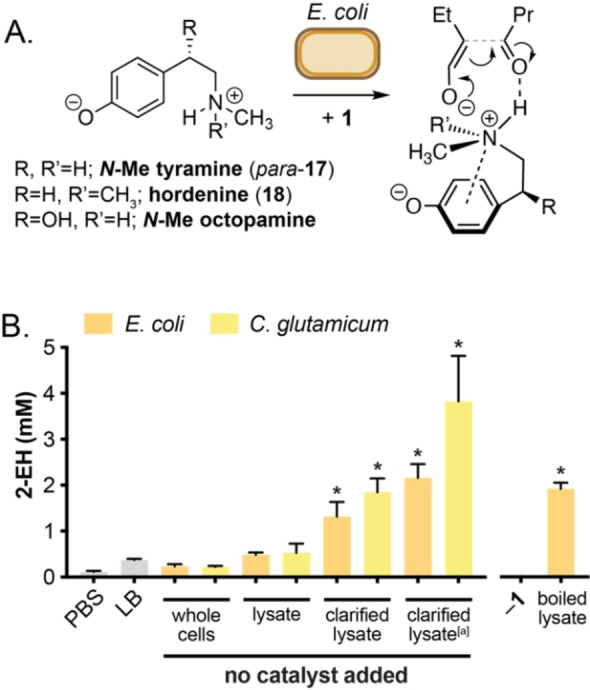
Mechanistic hypothesis and examining the catalyst‐free aldol reaction in the presence of intracellular amines generated by *E. coli* MG1655(K‐12) RARE and *C. glutamicum* ATCC13032. A) Proposed reaction mechanism. B) Screen using bacterial cell lysate. Butanal was added at 25 mM and reactions were incubated at 30 °C, pH 7.4 with shaking at 220 rpm for 24 h. Product concentrations in culture extracts were determined by ^1^H NMR spectroscopy relative to an internal standard of 1,3,5‐trimethyoxybenzene. All data are shown as an average of three independent experiments to one standard deviation. [a] pH 9.0. * P<0.05.

We then decided to compare the results obtained for biogenic amine catalysts in *E. coli* with synthetic amine organocatalysts – in particular those reported to catalyze the aldol reaction or similar C−C bond formations in aqueous solvent. To date, the reactions enabled by these modern organocatalysts have not been studied in the presence of a living microorganism. We selected a series of L‐proline derivatives including 2‐methoxymethyl pyrrolidine **12**, pyrrolidinylmethyl pyrrolidine **23**, proline tetrazole **21** and a L‐proline β‐naphthylamide **22** (Figure 3 and Table S2). These catalysts have been shown to catalyze a range of C−C bond formations, including enantioselective aldol reactions under aqueous reaction conditions.^[9]^ In particular, tetrazole **21** has been reported to catalyze the aldol modification of recombinant proteins in phosphate buffer and cell lysate at 37 °C.^[10]^ Alongside these, we also selected the cinchona alkaloids **19**/**20** and the MacMillan 2^nd^ Generation imidazolidinone catalyst **24**. These were screened alongside the transition metal complexes ferric ammonium citrate and bidentate Zn complexes of L‐Lys, L‐Arg and L‐Pro in order to assess whether the formation of **2**/**3** was being catalyzed by a transient active organometallic species generated *in situ* from trace metals present in the growth media or cell interior. To our surprise, the majority of these catalysts were inactive in the presence of *E. coli* cells. Cinchonidines **19**/**20** were particularly insoluble in cell culture and remained inactive when pre‐dissolved in DMSO or in the presence of a 10 % *n*‐dodecane overlay (Table 1, entries 25, 26, and Table S1). Interestingly, however, pyrrolidinylmethyl pyrrolidine **23** was active and comparable to many biogenic amines, producing 2‐EH **2** in 30 % yield (Table 1, entry 28). Product formation was reduced to 24 % using the methyoxymethyl prolinol analogue **12** indicating that the tertiary amine side‐chain of **23** enhanced activity *in vivo*. This aligns with our observations for hydroxy‐phenethylamines **14**–**18** and taken together suggests that a basic side‐chain bonded to a hydrogen bond donor motif is required for aldol activity in *E. coli*. Interestingly, we synthesized the isomeric *meta*‐ and *ortho*‐*N*‐Me tyramine catalysts (*meta*‐/*ortho*‐**17**) in two steps from the corresponding methoxyphenethyl bromide (Supporting Information Section S8) and found that the *ortho*‐isomer was less active and the *meta*‐isomer decreased product conversion to 18 % (Table 1, entries 13 and 14). This led us to hypothesize that the reaction proceeds via the initial deprotonation of the phenol to generate an inductively‐stabilized ammonium phenoxide, which then facilitates formation of the substrate enolate and aldol product by hydrogen bond donation from a cation‐π‐stabilized ammonium species (Figure 4A). This non‐covalent interaction is known to favor a ‘twisted’ configuration in tyramines at physiological pH and computational simulations indicate that this interaction is most prominent in the *para*‐tyramine isomer.^[11]^ This ammonium enolate mechanism has also been recently proposed by Hayashi *et al*. for the 2°‐amine catalyzed Michael reaction of α,β‐unsaturated aldehydes.^[12]^

We next moved on to assess the biocompatibility of phenethylamine catalysts to *E. coli. N*‐Methyl tyramine **17** and hordenine **18** inhibited growth (OD_600_) and cell viability (cfu/mL) at 25 mM. Tyramine **14** and *N*‐methyloctopamine decreased the number of viable cells by 10^4^‐10^5^‐fold at this concentration. Intriguingly, octopamine was remarkably biocompatible, resulting in no significant reduction in viable cells or OD_600_ growth when **15** was added to cultures of *E. coli* (Figures S9‐S11). Although the precise reason(s) for this biocompatibility are currently unclear, a stabilizing intramolecular hydrogen bond between the benzylic hydroxyl group and the terminal amine in **15** would disfavor ammonium cation formation in solution at pH 7–8 and preclude any disruptive ionic interactions with the negatively charged bacterial cell membrane.

Having shown that small organic amines can mediate the self‐aldol dimerization of butyraldehyde in the presence of living *E. coli*, we moved on to test whether endogenous amines produced in a living cell could have a similar effect. To do this we chose the bacterium *Corynebacterium glutamicum* ATCC 13032 – a Gram‐positive microorganism that is used for the industrial production of many platform amines and basic amino acids. Under standard growth conditions, amines such as L‐lysine in engineered *C. glutamicum* can exceed 30 mM.^[13]^ This was therefore the ideal system to test whether endogenous amines produced *in vivo* can mediate the biocompatible aldol reaction of an exogenous substrate. The intracellular concentration of free amino acids in *E. coli* is >110 mM; glutamate accounts for 96 mM, whereas the catalytically active amino acids arginine, lysine and proline are present in the cytoplasm at 0.57 mM, 0.41 mM and 0.39 mM, respectively.^[14]^ Therefore, we reasoned that cell lysate produced from high OD cultures of both microorganisms could be used as a source of amine catalysts for the self‐aldol dimerization of **1**. To this end, we observed minimal product formation with whole cells (*E. coli* or *C. glutamicum*, OD_600_=5.0) or without cells in LB or PBS buffer (<3 % yield, Figure 4B). This was increased to 10–15 % yield in the presence of supernatant isolated from spent cultures. Incubating butanal with lysates generated via sonication of cells re‐suspended to OD_600_ = 50 produced 1‐butanol in >95 % conversion (Section S6). However, product conversion was increased from 4 % to 18 % and 31 % yield for *E. coli* and *C. glutamicum*, respectively, by using only the soluble lysate fraction as a source of amine catalysts at pH 9. The presence of hydrophobic cell membrane lipids therefore plays a negligible role in the amine catalyzed aldol reaction of butanal in the presence of bacterial cells (logP_octanol/water_ 0.9 for **1**; logP _octanol/water_ −4.2 for Arg, −3.0 for Lys and −2.5 for Pro), unlike other reported biocompatible reactions.^[5]^ The near‐quantitative reduction of **1** in crude cell lysate also suggests that the aldol reaction is an extracellular process and that the competing reduction of butanal to 1‐butanol occurs enzymatically at the cytoplasmic or periplasmic membrane. A range of C3‐C6 aliphatic aldehyde substrates could be dimerized under these optimized conditions using lysates and/or **15**, affording the corresponding aldol and C=C reduced products in the presence of *E. coli* RARE (Table S4 and Figure S8). No product formation was observed by ^1^H NMR in the absence of substrate or cells, and was retained in the presence of boiled lysate samples, confirming that the observed reactivity in cell lysate was non‐enzymatic (Figure 4B). Together, these results not only demonstrate that cell lysate can be used as a source of amine organocatalysts for aldol chemistry *in vitro*, but also suggest that non‐enzymatic aldol reactions could be catalyzed by endogenous amines in living cells throughout Nature. For example, aldehydes, including enals, are known to act as semiochemicals to modulate the behavior of insects and plants. Intriguingly, 2‐EH and other enals have been detected in the wax‐lined Dufour gland of the *Camponotus* and *Apterostigma* ants and no experimental data exists to confirm that it is synthesized by endogenous enzymes. Invertebrates such as ants also use tyramine derived neurotransmitters, particularly octopamine and synephrine in this region.^[15]^ This observation suggests that organocatalytic amine‐catalyzed aldol chemistry could exist in Nature as an evolved strategy for the formation of diverse enals from simple aldehydes *in vivo*.

## Conclusion

To conclude, this study reports that biogenic amine organocatalysts derived from L‐tyrosine catalyze the self‐aldol dimerization of butyraldehyde in the presence of *E. coli* cells. These amine catalysts are biocompatible, present in living cells and therefore can be overproduced via metabolic engineering. In bacterial cell culture, these biogenic organocatalysts outperformed amine catalysts that have been used to label proteins *in vitro* and for diverse applications in aqueous and organic solvents in synthetic organic chemistry. Concentrated cell lysates containing amines from *E. coli* and *C. glutamicum* could also be used as a source of organocatalysts, demonstrating the potential for microbial cells to be used as reagents for green chemical reactions and hinting at the prevalence of non‐enzymatic organocatalysis in Nature. Future work from our laboratory in this area will include the cellular synthesis of these organocatalysts for use *in vitro* and *in vivo* and the application of organocatalytic reactivity within metabolic pathways for the sustainable synthesis of non‐natural products in a microbial host.

## Experimental Section

Full experimental details can be found in the Supporting Information.

## Author Contributions

S.W. and J.A.D. conceived of the project. J.A.D. performed and analyzed the experiments. J.C.S. synthesized catalysts. S.W. and J.A.D. wrote the manuscript.

## Conflict of interest

The authors declare no conflict of interest.

1

## Biographical Information

*Jonathan obtained a BSc in Chemistry with Pharmaceutical Chemistry from Heriot‐Watt University in 2017, and an MSc in Synthetic Biology & Biotechnology from the University of Edinburgh in 2018. The same year he joined the Wallace Lab at the University of Edinburgh to pursue his PhD as part of the EPSRC Centre for Doctoral Training in Critical Resource Catalysis (CRITICAT). He is currently a 4th year PhD candidate working on the development of biocompatible organocatalytic reactions that can be interfaced with living cells and cellular metabolism*.

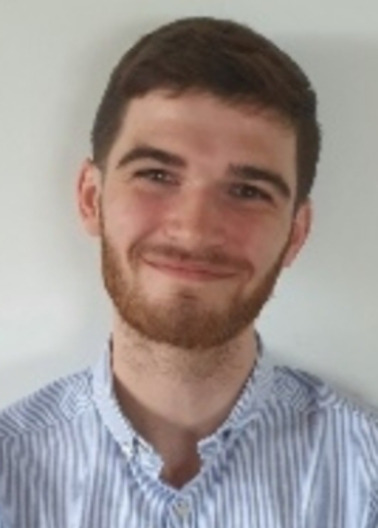

## Biographical Information

*Joanna obtained a MSc in Chemistry from the University of Bristol in 2013 and a PhD in Biocatalysis & Organic Chemistry from GlaxoSmithKline and the University of Strathclyde in 2017. She has held postdoctoral positions at the University of Manchester and the University of St Andrews before joining the Wallace Lab at the University of Edinburgh as a BBSRC Discovery Fellow from 2019–2020, where she worked on biocompatible chemistry and bio‐based plastic upcycling. She is now a Chancellor's Fellow in the School of Biological Sciences at the University of Edinburgh*.

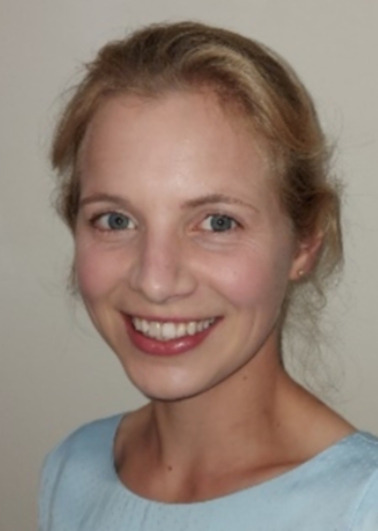

## Biographical Information

*Stephen is a UKRI Future Leaders Fellow and Senior Lecturer in Biotechnology in the School of Biological Sciences at the University of Edinburgh. He obtained a MChem in Medicinal and Biological Chemistry with Industrial Experience from the University of Edinburgh in 2008 and a DPhil in Organic Chemistry from the University of Oxford in 2012. He has held postdoctoral research fellowships at the MRC Laboratory of Molecular Biology, Harvard University and the University of Cambridge. His independent research spans the study and manipulation of microbial chemistry for use in sustainable chemical synthesis*.

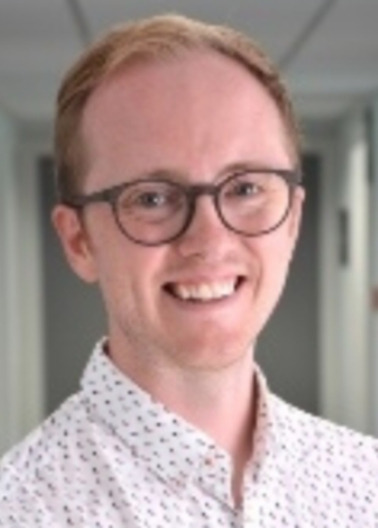

## Supporting information

As a service to our authors and readers, this journal provides supporting information supplied by the authors. Such materials are peer reviewed and may be re‐organized for online delivery, but are not copy‐edited or typeset. Technical support issues arising from supporting information (other than missing files) should be addressed to the authors.

Supporting Information

## Data Availability

The data that support the findings of this study are available in the supplementary material of this article.

## References

[1a] J. C. Sadler , J. A. Dennis , N. W. Johnson , S. Wallace , RSC Chem. Biol. 2021, 2, 1073–1083, https://pubs.rsc.org/en/content/articlelanding/2021/cb/d1cb00072a#!divAbstract; 34458824 10.1039/d1cb00072a PMC8341791

[1b] K. N. Stewart , D. W. Domaille , ChemBioChem 2021, 22, 469–477; 32851745 10.1002/cbic.202000458

[1c] S. Wallace , E. P. Balskus , Curr. Opin. Biotechnol. 2014, 30, 1–8. 24747284 10.1016/j.copbio.2014.03.006 PMC4199929

[2] J. Guo , M. Suástegui , K. K. Sakimoto , V. M. Moody , G. Xiao , D. G. Nocera , N. S. Joshi , Science 2018, 362, 813–816. 30442806 10.1126/science.aat9777 PMC6290997

[3] J. Liu , S. H. J. Chan , T. Brock-Nannestad , J. Chen , S. Y. Lee , C. Solem , P. R. Jensen , Metab. Eng. 2016, 36, 57–67. 26969254 10.1016/j.ymben.2016.02.008

[4] R. V. Masskant , S. Chordia , G. Roelfes , ChemCatChem 2021, 13, 1607–1613.

[5a] S. Wallace , E. P. Balskus , Angew. Chem. Int. Ed. 2016, 55, 6023–6027; 10.1002/anie.201600966 PMC4973394 27061024

[5b] S. Wallace , E. P. Balskus , Angew. Chem. Int. Ed. 2015, 54, 7106–7109; 10.1002/anie.201502185 PMC4494747 25925138

[6a] D. W. Domaille , G. R. Hafenstine , M. A. Greer , A. P. Goodwin , J. N. Cha , ACS Sustainable Chem. Eng. 2016, 4, 671–675; 28480149 10.1021/acssuschemeng.5b01590 PMC5417690

[6b] K. N. Stewart , E. G. Hicks , D. W. Domaille , ACS Sustainable Chem. Eng. 2020, 8, 4114–4119;

[6c] K. N. Stewart , D. W. Domaille , React. Chem. Eng. 2022, 7, 1328–1334.

[7] A. M. Kunjapur , Y. Tarasova , K. L. J. Prather , J. Am. Chem. Soc. 2014, 33, 11644–11654. 10.1021/ja506664a 25076127

[8] R. C. Brewster , J. T. Suitor , A. W. Bennett , S. Wallace , Angew. Chem. Int. Ed. 2019, 58, 12409–12414; 10.1002/anie.201903973 31286626

[9] H. Torii , M. Nakadai , K. Ishihara , S. Saito , H. Yamamoto , Angew. Chem. Int. Ed. 2004, 43, 1983–1986; 10.1002/anie.200352724 15065280

[10] R. J. Spears , R. L. Brabham , D. Budhadev , T. Keenan , S. McKenna , J. Walton , J. A. Brannigan , A. Marek Brzozowski , A. J. Wilkinson , M. Plevin , M. A. Fascione , Chem. Sci. 2018, 9, 5585–5593. 30061990 10.1039/c8sc01617h PMC6049525

[11] C. Cabezas , A. Simão , C. Bermúdez , M. Varela , I. Peña , S. Mata , R. Fausto , J. L. Alonso , J. Phys. Chem. A 2013, 117, 4907–4915. 23675821 10.1021/jp4032223

[12] N. Umekubo , T. Terunuma , E. Kwon , Y. Hayashi , Chem. Sci. 2020, 11, 11293–11297. 34094371 10.1039/d0sc03359f PMC8162273

[13] J. A. da Luz , E. Hans , D. Frank , A.-P. Zeng , Eng. Life Sci. 2017, 17, 512–522. 32624795 10.1002/elsc.201600163 PMC6999343

[14] B. D. Bennett , E. H. Kimball , M. Gao , R. Osterhout , S. J. Van Dien , J. D. Rabinowitz , Nat. Chem. Biol. 2009, 5, 593–599. 19561621 10.1038/nchembio.186 PMC2754216

[15a] C. T. Hogan , T. H. Jones , M. Zhukova , J. Sosa-Calvo , R. M. M. Adams , Biochem. Syst. Ecol. 2017, 72, 56–62;

[15b] H. L. Voegtle , T. H. Jones , D. W. Davidson , R. R. Snelling , J. Chem. Ecol. 2008, 34, 215–219. 18213494 10.1007/s10886-008-9430-6

