# Supplementary material for: Tyramine Derivatives Catalyze the Aldol Dimerization of Butyraldehyde in the Presence of Escherichia coli
Source: Chembiochem. 2022 Jul 14;23(17):e202200238. doi: 10.1002/cbic.202200238 (PMC9540883; doi:10.1002/cbic.202200238)
Supplement: Supplementary file 1 — Supporting Information [file CBIC-23-0-s001.pdf]

ChemBioChem

Supporting Information

Tyramine Derivatives Catalyze the Aldol Dimerization of Butyraldehyde in the Presence of *Escherichia coli*

Jonathan A. Dennis, Joanna C. Sadler, and Stephen Wallace*

**Page**	**Section**	**Contents**
**S3**	S1	General Methods
**S3**	S1.1	General materials and methods
**S4**	S1.2	Media, strains and culturing conditions
**S5**	S1.3	Butanal toxicity screen
**S6**	S1.4	Investigating the mass balance of butanal added to bacterial cultures
**S7**	S1.5	Catalyst screening reactions
**S8**	S2	GC-FID quantification methods
**S10**	S3	Catalyst screening data
**S15**	S4	*N*-methyltyramine isomers and control compounds
**S16**	S5	Substrate scope
**S17**	S6	Putative ene-reductases in *E. coli* MG1655
**S18**	S7	Cell lysate screening experiments
**S19**	S8	Measuring the toxicity of tyramine derivatives
**S21**	S9	Synthesis
**S23**	S10	References

S1 General Methods

S1.1 General Materials and Methods

E. coli MG1655 RARE cells were obtained from Addgene. *E. coli* MG1655 cells and plasmid pET22b were obtained from Prof. Chris French (University of Edinburgh). *Corynebacterium glutamicum* ATCC 13032 cells were obtained from Ingenza Ltd. (UK) All cells were stored as 1:1 v/v LB:glycerol stocks at −80 °C and used as required. Optical densities of *E. coli* and *C. glutamicum* cultures were determined using a DeNovix DS-11 UV/Vis spectrophotometer by measuring absorbance at 600 nm.

Proton nuclear magnetic resonance (NMR) spectra were aquired using a Bruker AVA 500 or PRO 500 (500 MHz) NMR spectrometer at 20 °C. Proton chemical shifts are expressed in parts per million (ppm, ∂ scale) and are referenced to residual protium in the NMR solvent ($CDCl_3$, ∂ 7.26 ppm). Coupling constants, *J*, are measured to the nearest 0.1 Hz and are presented as observed. Data is represented as: chemical shift, integration, multiplicity (s = singlet, d = doublet, t = triplet, q = quartet, dd = doublet of doublet, m = multiplet and/or multiple resonances), coupling constant (*J*) in Hertz.

All chemicals and solvents were purchased from commercial suppliers and were used without further purification. All catalysts used in the initial screen were purchased from Sigma Aldrich or Fluorochem UK. The *ortho*- and *para*- isomers of *N*-methyltyramine were respectively synthesised in two steps from 2- and 3-methoxyphenylethyl bromide according to a known procedure[2], outlined in S8. All water used experimentally was purified with a Suez Select purification system (18 mΩ cm^{-1}, 0.2 µm filter). All GC and NMR solvents were purchased from Sigma Aldrich. For all quantitative measurements by ^{1}H-NMR spectroscopy or GC-FID, 1,3,5-trimethoxybenzene (TMB, 3 mM) was used as an internal standard.

S1.2 Media, Strains and Culturing Conditions

Luria Bertani Lennox (LB) Media was prepared according to the following procedure: bacto-tryptone (10 g/L), yeast extract (5 g/L) and NaCl (10 g/L) was dissolved in ultrapure H_2O. LB was autoclaved at 121 °C for 20 min, cooled and stored at room temperature. LB agar was made using the same recipe but with the addition of agar (15 g/L). SOC media was prepared according to the following procedure: bacto-tryptone (20 g/L), yeast extract (5 g/L) and NaCl (0.5 g/L) were dissolved in ultrapure H_2O. KCl was added to a final concentration of 2.5 mM. The mixture was autoclaved at 121 °C for 20 min and cooled before adding glucose (20 mM) and $MgCl_2$ (10 mM). SOC media was then stored at room temperature.

All other reagents used in culturing experiments were filter sterilised using a 0.22 µm filter (Millex). All chemically competent cells were prepared via treatment with calcium chloride [1]. For use as a culture inoculum, chemically competent *E. coli* MG1655 or MG1655 RARE were prepared and transformed with a pET22b(+) plasmid (Amp^r) via heat-shock at 42 °C for 45 s. Cells were recovered in 1 mL of SOC media for 1 h at 37 °C. Transformants were selected by plating on LB agar containing appropriate antibiotics and incubating at 37 °C overnight. A single colony was picked and grown overnight in 10 mL of LB containing appropriate antibiotics. The resulting overnight culture (0.5 mL) was added to 0.5 mL of 1:1 v/v water:glycerol solution, frozen in liquid nitrogen, stored at –80 °C and used as required. For microbiological experiments, antibiotics were used at the following concentrations: ampicillin (Amp), 100 µg/mL.

E. coli cultures were prepared by inoculating 10 mL of LB with a –80 °C LB:glycerol stock of *E. coli* MG1655 RARE_pET22b(+) or *E. coli* MG1655_pET22b(+) and incubating cultures at 30 °C (220 rpm) for 18 h. The saturated overnight culture (5 mL) was then inoculated into LB (250 mL in 500 mL Erlenmeyer flask) containing ampicillin and grown aerobically at 220 rpm until the culture reached OD_{600} = 0.5−0.6 (ca. 2.5−3 h). *E. coli* MG1655 was grown at 37 °C, and *E. coli* RARE was grown at 30 °C.

C. glutamicum WB2 cultures were prepared by inoculating 10 mL of LB with a –80 °C LB:glycerol stock of *C. glutamicum* WB2, and incubating at 30 °C (220 rpm) for 18 h. The saturated overnight culture (5 mL) was then inoculated into LB (250 mL in 500 mL Erlenmeyer flask) and grown aerobically at 30 °C (220 rpm) until the culture reached OD_{600} = 0.5−0.6 (ca. 2.5−3 h).

Cell lysates were prepared according to the following procedure: using a fresh overnight culture of the bacterium, 1 L LB media in a 2 L baffled flask containing appropriate antibiotics was inoculated (1:100) and grown for 24 h at the relevant temperature reaching an OD_{600} value of 3.0−7.0. Cells were collected using centrifugation (10,000 × *g*, 10 min, 4 °C). The supernatants were collected, and a sample taken for use as a media/catalyst source for screening reactions. The cell pellets were resuspended in ice-cold

Phosphate Buffered Saline (PBS) to a cell density of OD_{600} = 50. Keeping samples on ice throughout, an ultrasonic cell disruptor was used to lyse the cells (50% amplitude, 30 seconds on/off, 10 min total). Half of the crude lysate was centrifugated (20 min, 24,000 × *g*, 4 °C). The crude lysate and resulting supernatant (clarified lysate), were used as reaction media/catalyst source for screening reactions.

S1.3 Butanal Toxicity Screen

A fresh 250 mL culture of *E. coli* MG1655 or MG1655 RARE was grown to OD 0.5−0.6 and 7.5 mL of the resulting culture was added to sterile Hungate tubes. Using a gas tight syringe, a range of butanal concentrations (0.1−100 mM) were added to the cultures. The tubes were then sealed using butyl rubber septa and incubated horizontally at 30 °C, 220 rpm. After 24 h, 100 µL of each reaction mixture was removed and added to 900 µL of MQ water. These aliquots were subjected to serial 10-fold dilutions (10^1–10^8). Aliquots (100 µL) of each dilution were plated onto individual LB agar plates containing ampicillin (100 µg/mL). The plates were incubated at 30 °C overnight and the number of colonies from these plates were used to calculate the number of colony-forming units (CFUs) in each mL of culture.

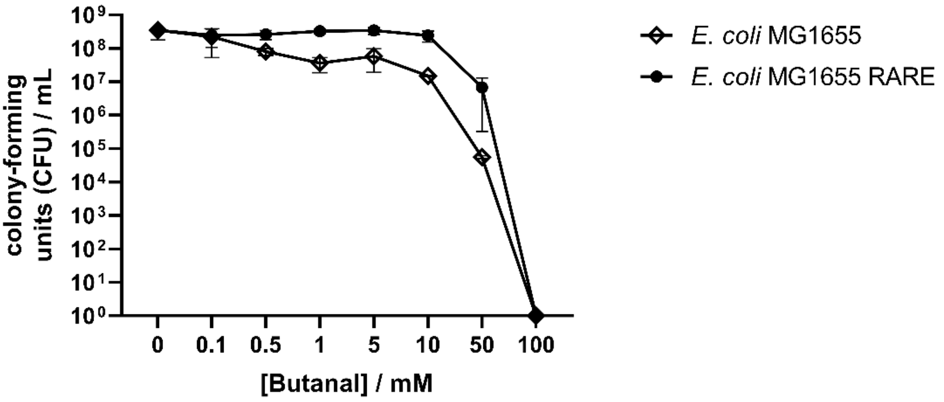

Figure S1: CFU assay comparing the effects of butanal (0.1−100 mM) on the viability of *E. coli* MG1655 and *E.coli* MG1655 RARE. Error bars represent the standard deviation of experiments performed in triplicate as outlined above.

S1.4 Investigating the mass balance of butanal added to bacterial cultures

Butanal (25 mM) was added to a Hungate tube containing 7.5 mL of a fresh culture (OD = 0.5−0.6) of *E. coli* MG1655 RARE or *C. glutamicum* WB2. A control sample was prepared by replacing the bacterial culture with sterile LB medium. The tubes were sealed and incubated horizontally for 24 h at 30 °C, 220 rpm. The contents of the tubes were extracted using the protocol for catalyst screening reactions. The concentration of butanal, butanol and 2-ethyl-2-hexenal in the samples was determined by ^{1}H NMR.

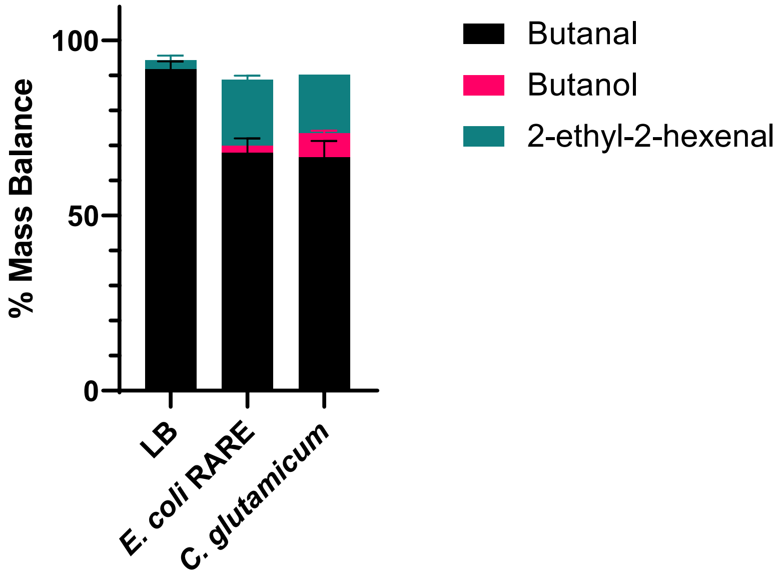

Figure S2: Mass balance recovery of samples in LB media or bacterial cultures. Error bars represent the standard deviation of triplicate samples quantified using ^{1}H NMR as outlined in S1.5.

S1.5 Catalyst Screening Reactions

All catalyst screening reactions were carried out in triplicate using autoclaved 15 mL glass Hungate tubes with butyl rubber septa and screw caps. Tubes contained 7.5 mL reaction volume and 7.5 mL headspace. Reaction media/cultures were preadjusted to the desired pH using either hydrochloric acid (2 M) or $NaOH_{(aq)}$ (2 M). Catalysts were weighed directly into the empty tubes, to which 7.5 mL reaction media was added. Adding catalysts after adjusting the pH to 9.0 resulted in small changes to the pH (< 0.25). Butanal was then added using a gas-tight syringe.

The Hungate tubes were then incubated for 24 h (30 °C, 220 rpm), after which they were cooled to −20 °C for 15 min. Aliquots (0.5 mL) were added to an equal volume of brine in 2 mL microcentrifuge tubes, to which diethyl ether (0.333 mL) containing 2 mM TMB was added. The tubes were vortexed for 3 min, then centrifugated for 2.5 min (4,500 × *g*, 4 °C). The organic phase was decanted into clean microcentrifuge tubes using a glass Pasteur pipette. This extraction process was repeated three times in total. Anhydrous sodium sulfate was added to dry the extract, which was then added to vials for GC-FID analysis. For ^1H NMR analysis, samples were extracted using $CDCl_3$ and analyzed directly.

S2 GC-FID Quantification Methods

Method 1: Gas chromatography was carried out using a Hewlett-Packard HP5890 GC instrument equipped with a Restek® Rxi-5ms, 5% diphenyl / 95% dimethylpolysiloxane column (30 m × 0.32 mmID. × 1 µm film). The GC inlet was maintained at 250 °C and the samples were injected in 1:5 split mode. The carrier gas was helium (1 mL/min). Samples were equilibrated for 3 min at 50 °C and then heated to 250 °C at a rate of 20 °C per min before the oven temperature was held at 250 °C for a final 1 min. The total run time was 13.0 min. Analytes were detected using a flame ionization detector (FID) with a heater temperature of 300 °C. For GC quantification, a standard curve was constructed over a range of analyte concentrations (0.5 mM – 100 mM) providing linear relationships of $pA_{butanal}/pA_{TMB} = 0.076c_{butanal}$, $pA_{butanol}/pA_{TMB} = 0.108c_{butanol}$, $pA_{2\text{-ethylhexanal}}/pA_{TMB} = 0.213c_{2\text{-ethylhexanal}}$, $pA_{2\text{-ethylhexenal}}/pA_{TMB} = 0.189c_{2\text{-ethylhexenal}}$ (Figure S4).

Method 2: Due to disruption caused by the Coronavirus pandemic, a different GC-FID system (Shimadzu GC-2010 Pro, equipped with a Restek® Rxi-5HT, 5% diphenyl / 95% dimethylpolysiloxane column (30 m × 0.32 mmID. × 1 µm film)) was used for some samples. All GC-FID gases were provided by gas generators from Peak Scientific (Scotland, UK). Hydrogen was used as FID fuel gas, nitrogen was used as the makeup gas, and hydrocarbon-free air was used as the oxygen source for the FID. The GC-FID inlet was maintained at 300 °C and samples were injected in 1:15 split mode. The carrier gas was hydrogen (2.33 mL/min). Samples were equilibrated for 3 min at 30 °C and then heated to 330 °C at a rate of 35 °C per min before the oven temperature was held at 330 °C for a final 1 min. The total run time was 12.6 min. Analytes were detected using a flame ionization detector (FID) with a heater temperature of 350 °C.

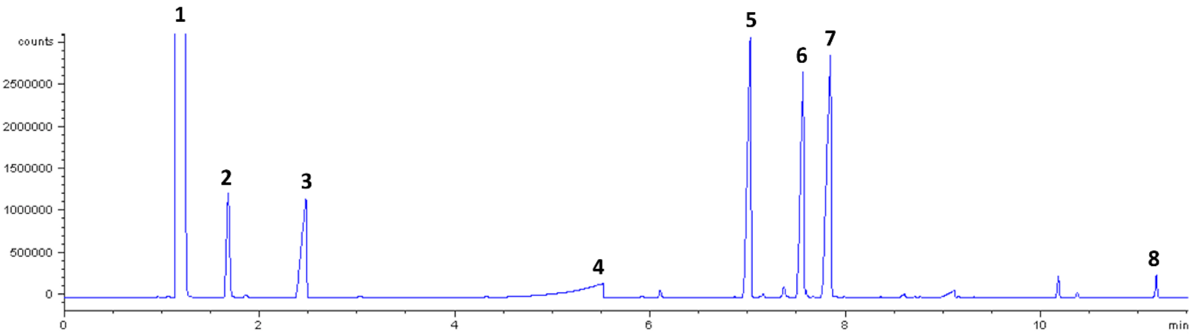

Figure S3: GC-FID trace of a synthetic mix of the reaction components. 1 = Et_2O, 2 = butanal, 3 = butanol, 4 = butyric acid, 5 = 2-ethylhexanal, 6 = 2-ethyl-2-hexenal, 7 = 2-ethyl-2-hexanol, 8 = TMB.

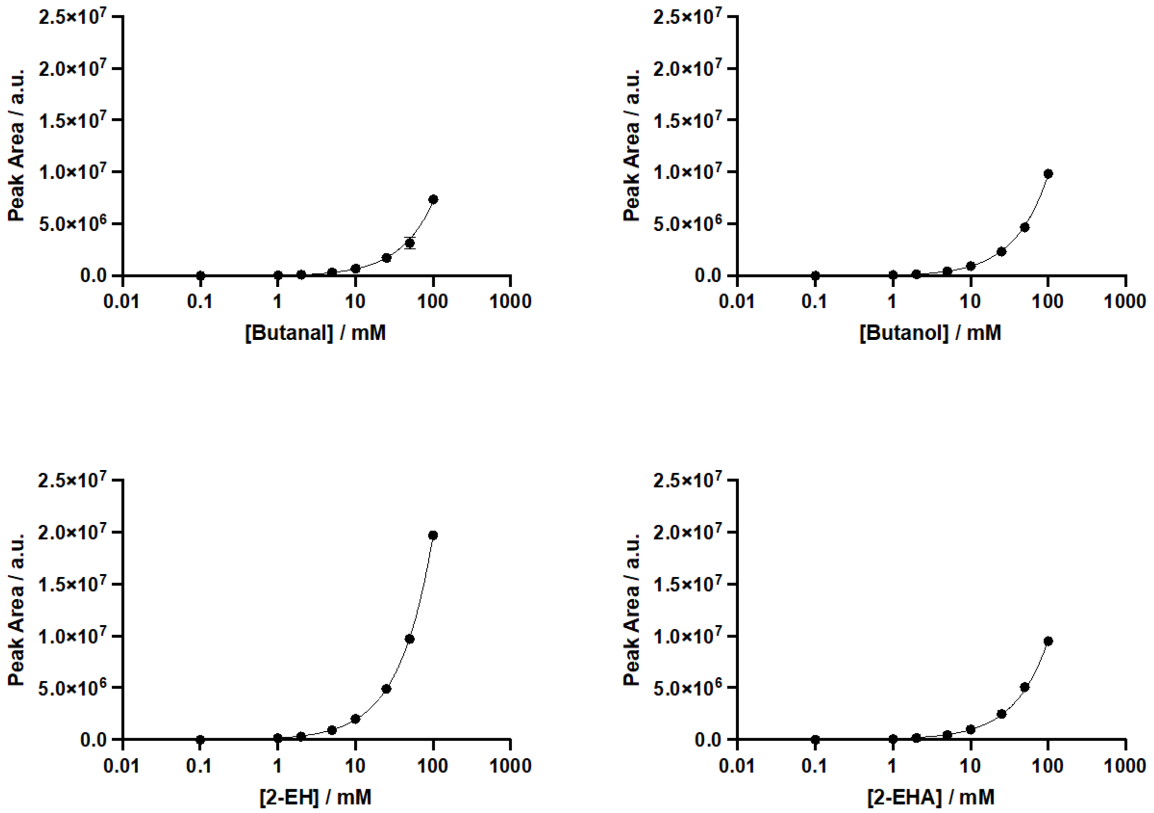

Figure S4: Calibration curves obtained for butanal, butanol, 2-EH and 2-EHA over a range of analyte concentrations (0.1−100 mM) in diethyl ether using the GC Method 2. The line of best fit was determined by linear regression on an exponential x-axis using GraphPad Prism. $r^2 > 0.99$ in all cases. All data points were obtained from triplicate samples.

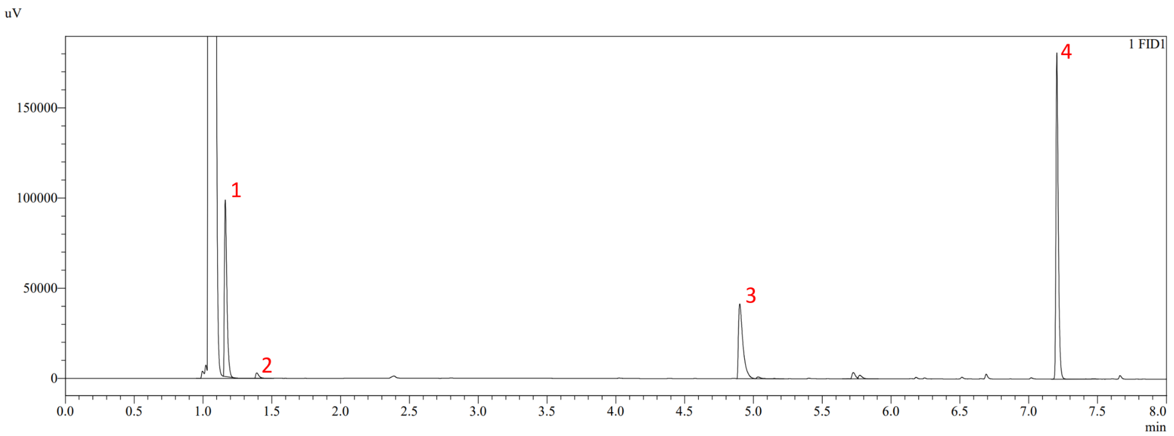

Figure S5: GC trace of an octopamine-catalyzed aldol dimerization reaction analyzed using GC-FID Method 2 (*E. coli* RARE + 25 mM butanal + 100 mol% octopamine, 24 h reaction at 30 °C). Peak numbers correspond as follows: 1 = butanal, 2 = butanol, 3 = 2-ethyl-2-hexenal, 4 = TMB.

S3 Catalyst Screening Data

Table S1: Results from screening compounds as catalysts, with ±SD representing the standard deviation of experiments performed in triplicate as outlined in S1.5.

amine	mol%	pH	butanal		Normalised§ 2-EH yield		Normalised§ 2-EHA yield	
			%	±SD	%	±SD	%	±SD
None	0	4	92	-	0 (4)	-	0 (0)	-
	0	7	90	-	0 (1)	-	0 (6)	-
	0	9	79	-	0 (13)	-	0 (2)	-
agmatine	100	9	90	2.5	14	1.4	4	0.12
ß-alanine	25	7	76	1.0	<1	0.03	1	0.06
	100	9	96	2.2	8	1.4	8	0.40
L-alanine	25	7	68	0.80	<1	0.01	2	0.03
6-aminocaproic acid	100	9	96	4.2	14	0.71	8	0.14
	25	7	68	5.5	<1	0.03	1	0.13
L-arginine	25	4	92	0.4	1	0.08	<1	0.03
	25	7	64	3.0	1	0.08	2	0.14
	100	4	96	4.6	1	0.32	<1	0.03
	100	7	48	0.72	12	1.1	<1	0.01
	100	9	52	2.0	33	2.7	<1	0.02
L-asparagine	25	7	64	6.6	<1	0.08	1	0.13
L-aspartic acid	25	7	76	1.5	1	0.05	1	0.01
cinchonidine	25[a]	9	60	2.6	4	0.16	<1	0.04
	25	9	56	0.20	3	0.80	<1	0.03
	25[b]	9	52	1.4	8	0.48	<1	0.02
cinchonine	100	9	60	9.2	4	3.0	<1	0.26
	25	9	56	1.5	5	1.2	1	0.18
creatinine	25	9	68	0.80	<1	0.01	1	0.04
D-cycloserine	100	9	48	2.1	1	0.71	<1	0.02
L-cysteine	25	7	56	2.5	<1	0.03	<1	0.01
cystine	25	7	64	2.1	<1	0.02	1	0.04
dopamine	25	7	56	3.0	1	0.96	**1**	0.80
GABA	25	7	72	1.3	<1	0.05	1	0.02
	100	9	96	2.6	7	0.71	8	0.12
L-glutamic acid	25	7	72	1.4	<1	0.02	1	0.03
L-glutamine	25	7	68	1.0	<1	0.02	2	0.07
glycine	25	7	72	2.1	4	0	1	0.06

histamine	25	7	72	4.0	<1	0.03	2	0.14
L-histidine	25	7	68	1.5	<1	0.02	2	0.07
hordenine	25	7	88	0.30	2	0.08	3	0.14
	25	9	44	0.52	21	0.64	0	0.00
	100	7	52	2.8	22	0.48	<1	0.04
	100	9	32	0.50	38	0.32	0	0.00
L-leucine	25	7	72	1.00	<1	0.01	2	0.04
L-lysine	25	4	88	3.5	<1	0.24	<1	0.05
	25	7	64	0.56	1	0.02	3	0.03
	25	9	44	2.5	22	3.9	<1	0.12
	100	4	96	1.8	1	0.16	<1	0.01
	100	9	52	2.1	26	3.2	1	0.09
L-methionine	25	7	68	0.88	<1	0.05	1	0.01
L-*N*(6)-methyl-lysine	25	9	32	5.8	27	2.2	<1	0.10
	100	9	32	7.5	35	1.6	<1	0.11
N-methyl octopamine	25	7	92	3.8	3	2.2	8	0.80
	100	9	52	6.00	27	1.6	<1	0.14
N-methyltyramine	25	7	96	6.4	8	2.5	3	0.33
	25	9	44	2.0	25	1.2	0	0.00
	100	7	60	0.80	21	3.9	<1	0.22
	100	9	24	0.88	37	2.5	<1	0.11
	100[c]	9	80	4.8	19	0.48	<1	0.00
octopamine	25	7	72	6.6	2	0.16	2	0.15
	100	9	68	0.92	25	1.4	2	0.06
L-ornithine	100	9	92	1.3	9	0.71	8	0.80
L-phenylalanine	25	7	68	4.2	<1	0.03	1	0.05
L-pipecolic acid	100	9	96	2.4	3	0	3	0.27
piperidine	100	9	64	4.4	23	0.64	0	0.00
L-proline	25	7	72	0.68	<1	0.01	1	0.05
	100	9	96	0.76	1	0.16	4	0.16
D-proline	25	7	68	3.8	<1	0.01	2	0.15
	100	9	92	9.9	1	0.24	4	0.36
pyroglutamic acid	100	9	76	4.8	<1	0.01	1	0.06
sarcosine	25	7	84	5.0	5	0.08	2	0.80
L-serine	25	7	68	1.40	<1	0.08	1	0.02
thiamine monochloride	25	7	72	0.76	<1	0.01	1	0.06
L-threonine	25	7	72	3.0	<1	0.08	1	0.22
trans-4-hydroxy-L-Proline	25	7	72	2.0	8	0.03	2	0.10

L-tryptophan	25	7	68	3.0	<1	0.02	1	0.09
tyramine	25	4	88	1.7	1	0.24	<1	0.02
	25	7	64	5.7	4	3.3	2	1.60
	25	9	36	2.6	14	0.72	0	0.00
	100	4	92	1.6	1	0.16	<1	0.06
	100	7	8	1.6	17	0.88	0	0.00
	100	9	24	2.3	19	0.96	<1	0.24
L-tyrosine	25	7	72	2.2	<1	0.02	1	0.09
uracil	25	7	68	3.1	<1	0.05	1	0.01
L-valine	25	7	72	<1	<1	0.02	1	0.10
L-alanine-ß-naphthylamide	100	9	104	0.64	2	0.10	4	0.15
(*S*)-1-amino-2-(methoxymethyl) pyrrolidine	100	9	72	0.12	<1	0.07	<1	0.03
casamino acids	-	9	72	1.7	1	0.01	1	0.05
cycloleucine	25	7	68	5.5	<1	0.03	1	0.12
N,N-dimethyl guanidine	100	9	112	6.0	7	0	2	0.16
(*2S,5S*)-(-)-2-^{t}Bu-3-Me-5-Bz-4- Imidazolidinone	25	9	100	2.5	2.1	0.61	2	0.29
(*S*)-(+)-2-methoxymethyl-pyrrolidine	100	9	60	1.2	24	0.93	0	0.00
N-methyl pyrrolidine	100	9	64	2.1	23	0.36	<1	0.04
L-norvaline	25	7	68	1.6	<1	0.02	2	0.12
L-proline-ß-naphthylamide	100	9	32	4.6	2	0.34	<1	0.00
(*S*)-5-(pyrrolidine-2-yl)tetrazole	25	9	88	1.8	1	0.15	5	0.28
	100	9	80	3.6	5	1.4	7	0.59
(*S*)-2-(pyrrolidinyl methyl) pyrrolidine	25	9	52	0.95	30	0.29	<1	0.18
L-terleucine	25	7	72	1.6	<1	0.01	1	0.05

§ Values normalised by subtracting negative control yield from sample yield. [a]= 10% (v/v) dodecane was added, [b]= 10% (v/v) DMSO was added, [c]= Reaction run at room temperature, n.d. = not determined. Numbers in parentheses represent the absolute yield.

Table S2: Structures of compounds screened. GABA = γ-aminobutyric acid. Unless otherwise stated, all compounds were screened in triplicate at 100 mol% loading with 25 mM exogenous butanal in the presence of *E. coli* MG1655 RARE (OD = 0.5−0.6) at pH 9.

<table>
<tr><th colspan="4">Biogenic Amines</th></tr>
<tr><td colspan="4">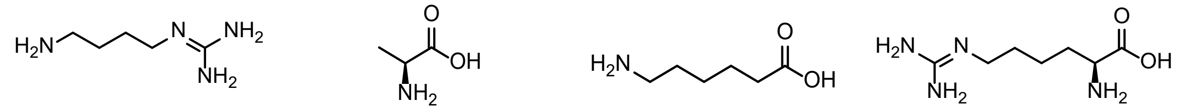</td></tr>
<tr><td>agmatine</td><td>L-alanine</td><td>6-aminocaproic acid</td><td>L-arginine</td></tr>
<tr><td colspan="4">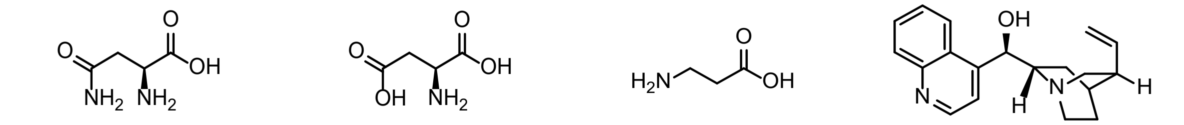</td></tr>
<tr><td>L-asparagine</td><td>L-aspartic acid</td><td>ß-alanine</td><td>cinchonidine</td></tr>
<tr><td colspan="4">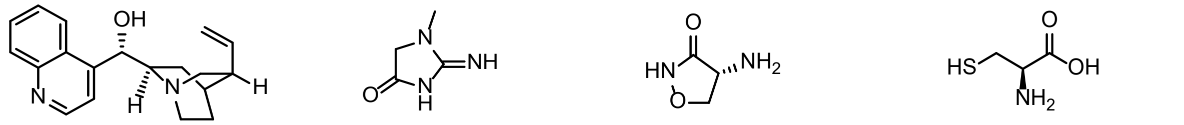</td></tr>
<tr><td>cinchonine</td><td>creatinine</td><td>D-cycloserine</td><td>L-cysteine</td></tr>
<tr><td colspan="4">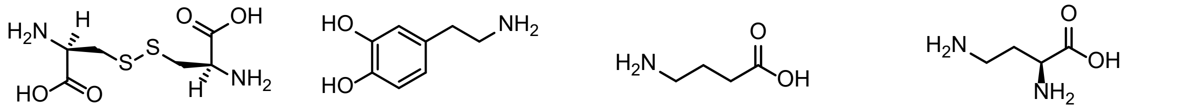</td></tr>
<tr><td>cystine</td><td>dopamine</td><td>GABA</td><td>L-glutamic acid</td></tr>
<tr><td colspan="4">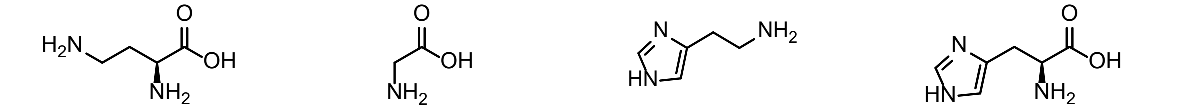</td></tr>
<tr><td>L-glutamine</td><td>glycine</td><td>histamine</td><td>L-histidine</td></tr>
<tr><td colspan="4">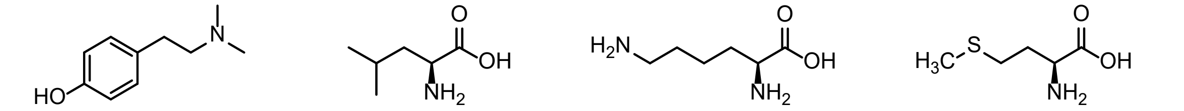</td></tr>
<tr><td>L-hordenine</td><td>L-leucine</td><td>L-lysine</td><td>L-methionine</td></tr>
<tr><td colspan="4">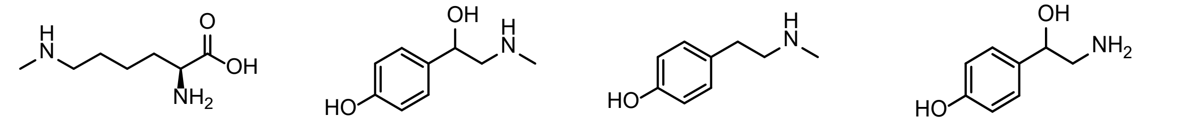</td></tr>
<tr><td>L-N(6)-methyl-lysine</td><td>N-methyl octopamine</td><td>N-methyltyramine</td><td>octopamine</td></tr>
<tr><td colspan="4">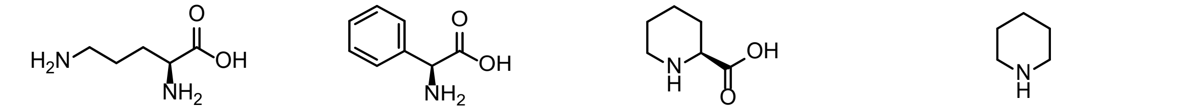</td></tr>
<tr><td>L-ornithine</td><td>L-phenylalanine</td><td>L-pipecolic acid</td><td>piperidine</td></tr>
<tr><td colspan="4">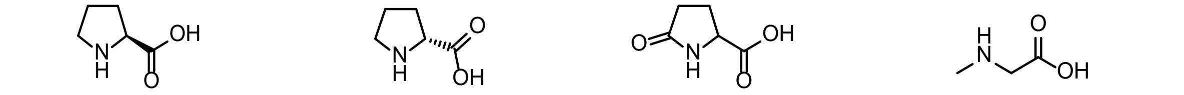</td></tr>
<tr><td>L-proline</td><td>D-proline</td><td>pyroglutamic acid</td><td>sarcosine</td></tr>
<tr><td colspan="4">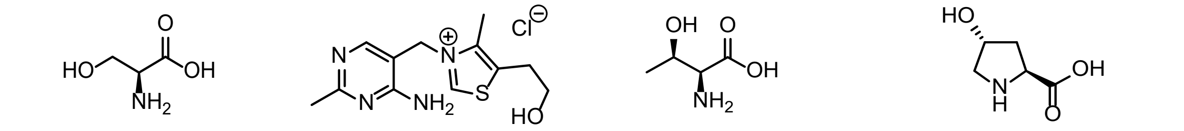</td></tr>
</table>

L-serine	thiamine chloride	L-threonine	*trans*-4-hydroxy-L-Proline
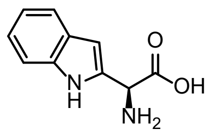	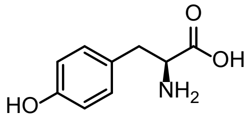	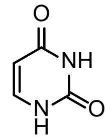	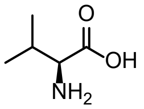
L-tryptophan	L-tyrosine	uracil	L-valine
**Synthetic Amines**			
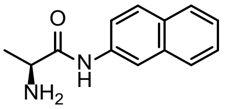	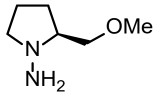	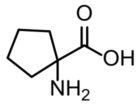	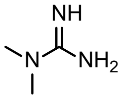
L-alanine-ß-naphthylamide	(*S*)-1-amino-2-(methoxymethyl) pyrrolidine	cycloleucine	*N,N*-dimethyl guanidine
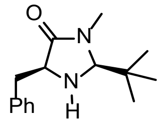	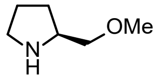	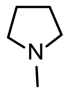	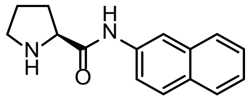
(*2S,5S*)-(-)-2-[t]Bu-3-Me-5-Bz-4-Imidazolidinone	(*S*)-(+)-2-methoxymethyl-pyrrolidine	*N*-methylpyrrolidine	L-proline-ß-naphthylamide
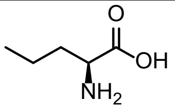	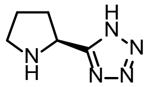	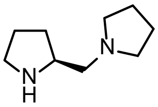	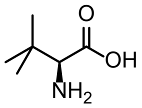
L-norvaline	(*S*)-5-(pyrrolidine-2-yl)tetrazole	(*S*)-2-(pyrrolidinyl methyl) pyrrolidine	L-terleucine

S4 *N*-methyltyramine isomers and control compounds

Table S3: Tyramine derivatives used to investigate the structure-function relationship of the catalytic activity of *N*-methyltyramines in the aldol condensation reaction of butanal. All experiments performed in triplicate as outlined in S1.5.

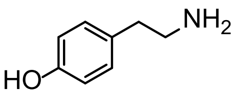	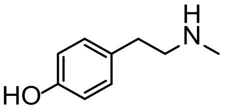	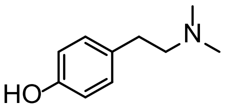	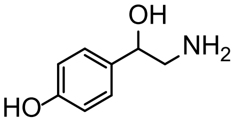
tyramine<br>19% yield	*N*-methyltyramine<br>35% yield	hordenine<br>38% yield	octopamine<br>25% yield
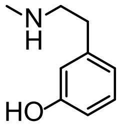	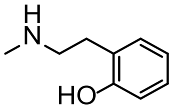	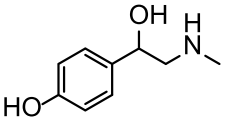	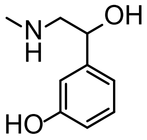
m-*N*-methyltyramine<br>18% yield	*o*-*N*-methyltyramine<br>22% yield	*N*-methyloctopamine<br>27% yield	*m*-octopamine<br>24% yield
	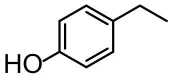	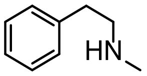	
	4-ethylphenol<br>15% yield	*N*-methyl<br>phenylethylamine<br>27% yield	

S5 Substrate scope

Table S4: Yields of aldol dimer and C=C reduced dimer obtained in the presence and absence of octopamine (25 mM). All experiments perfomed in triplicate as outlined in S1.5 substituting butanal for the relevant aldehyde starting material.

**Starting Material**	**[Octopamine] / mM**	**% Yield Aldol Dimer**	**% Yield C=C Reduced Dimer**
propanal	0	8	0
	25	46	0
pentanal	0	4	3
	25	12	1
3-methylbutanal	0	1	1
	25	1	1
hexanal	0	8	1
	25	10	0

S6 Investigating Putative Ene-reductases in *E. coli* MG1655

Table S5: Sequence alignment results from *Gluconobacter oxydans* enoate reductase (WP_011252080.1) aligned to the reference genome of *E. coli* MG1655

Hit #	Description	Query Cover	E Value	Identity	Accession No.	Gene ID
1	*N*-ethylmaleimide reductase [*Enterobacteriaceae*]	97%	5e-86	44%	WP_000093589.1	*nemA*
2	NADPH-dependent 2,4-dienoyl-CoA reductase [*Escherichia coli*]	94%	8e-27	29%	WP_166695766.1	*fadH*

A reference sequence of *Gluconobacter oxydans* enoate reductase (WP_011252080.1) was aligned against the *E. coli* K12 MG1655 reference genome using the NCBI BLAST blastp program[3] with default settings. Genes for putative ene-reductases in *E. coli* MG1655, identified by bioinformatics or enzymes with known ene-reductase activity, were knocked out by obtaining single-gene knockouts from the Keio collection on Addgene. Cultures of each strain were grown according to the protocol in S1.2, after which 7.5 mL of culture was added to sterile 15 mL Hungate tubes containing 2-ethyl-2-hexenal (1 mM). The cultures were then incubated horizontally at 30 °C for 24 h at 220 rpm. Samples were analysed by ^{1}H NMR as outlined in S1.5.

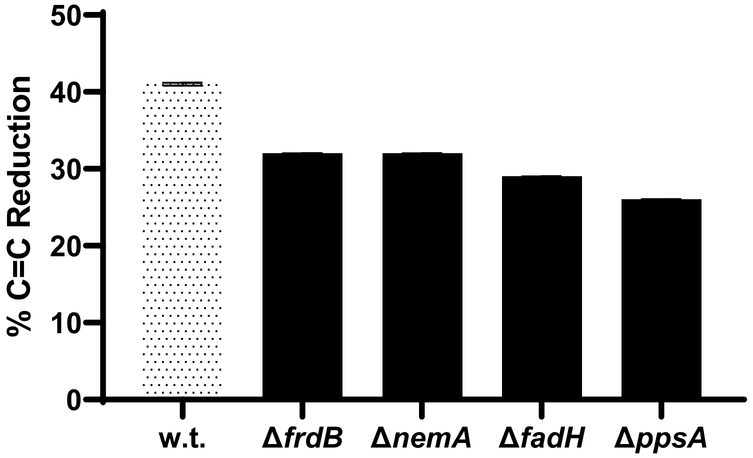

Figure S6: Reduction of 2-ethyl-2-hexenal by mid-log phase cultures of *E. coli* BW25113 and one of four gene knockouts

S7 Cell Lysate Screening Experiments

Cell lysates of *E. coli* MG1655 RARE and *C. glutamicum* were prepared as outlined in S1.2. Butanal (25 mM) was added to 7.5 mL samples of PBS, LB medium, OD=5 supernatants, crude lysates, clarified lysates and clarified lysates at pH 9. After 24 h incubation at 30 °C, samples were extracted and analysed by ^{1}H NMR spectroscopy as outlined in S1.5.

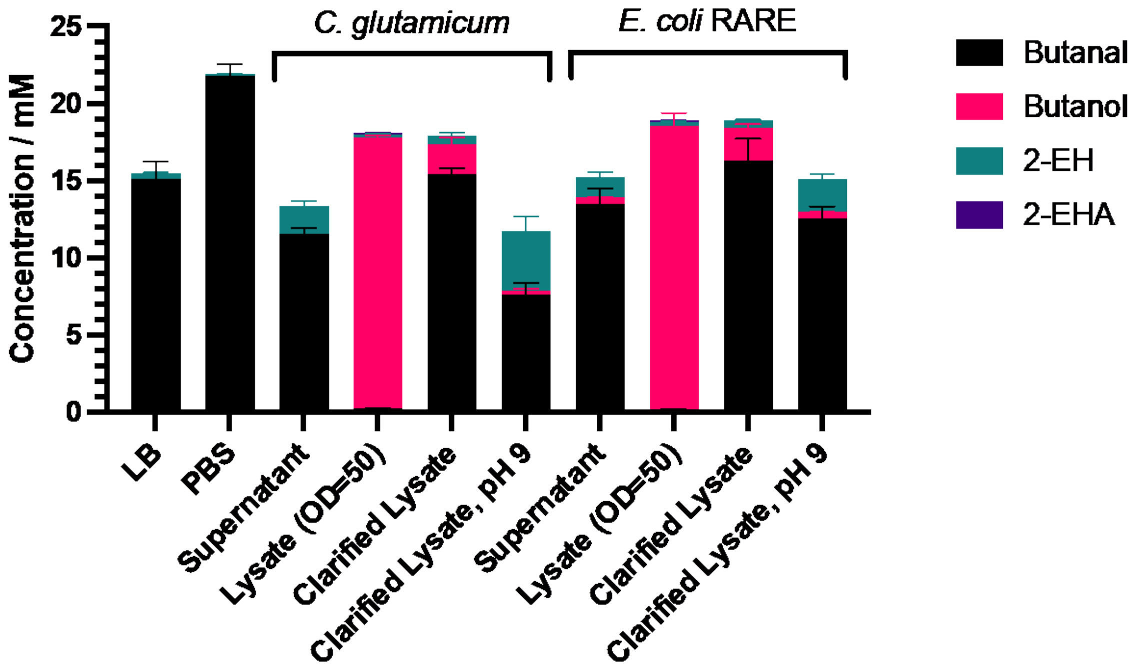

Figure S7: Mass balance of reactions involving cell lysates incubated with 25 mM butanal.

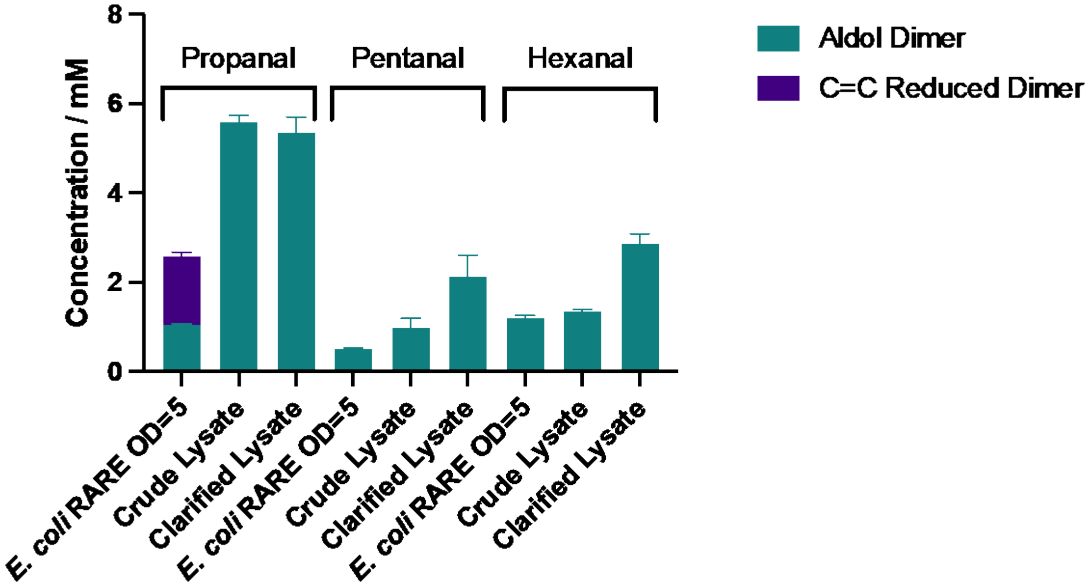

Figure S8: Yields of aldol dimer and corresponding C=C reduced product from propanal, pentanal and hexanal substrates (25 mM) in cultures or lysates of *E. coli* RARE at pH 9.0.

S8 Measuring the toxicity of tyramine derivatives

Mid-log cultures of *E. coli* MG1655 RARE were grown as outlined in S1.2 and added to sterile Hungate tubes containing 25 mM of tyramine derivative. The tubes were sealed with rubber septa and screw caps, then incubated at 30 °C (220 rpm). Optical density measurements were taken regularly for the first 4–5 h. After 24 h, serial dilutions were prepared as outlined in S1.3, and plated on ampicillin-containing agar plates before counting colonies for CFU measurements.

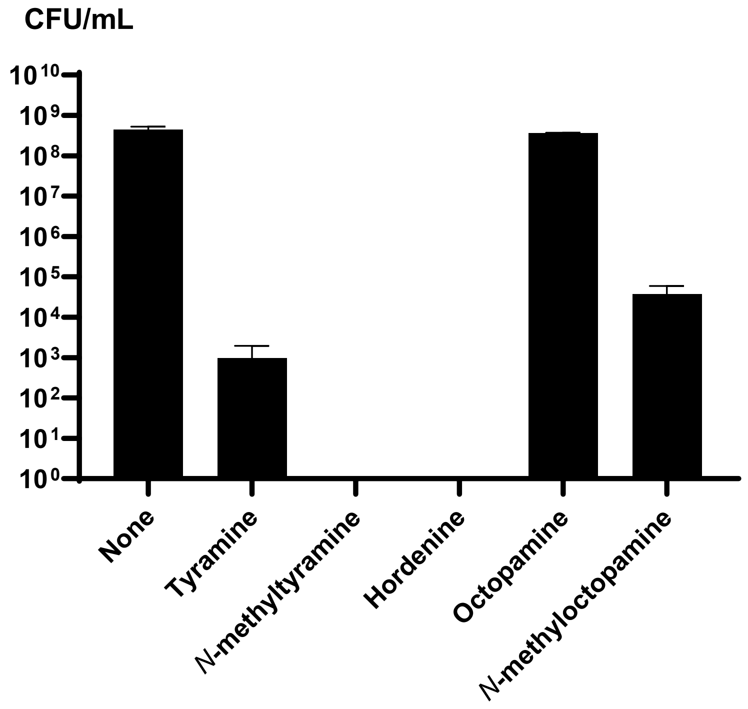

Figure S9: Comparing the effects of tyramine derivatives on the viability of *E. coli* MG1655 RARE (pH = 9)

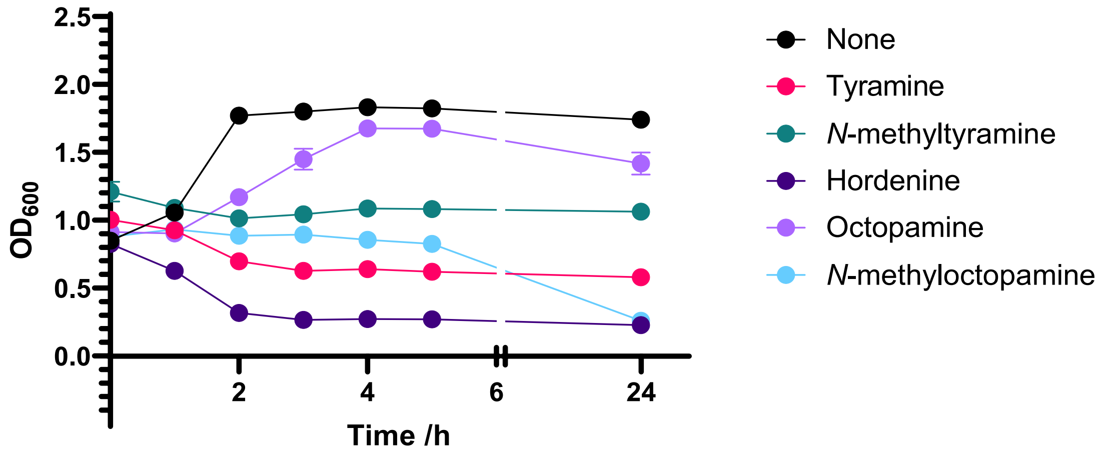

Figure S10: Comparing the effects of tyramine derivatives on the optical density of *E. coli* MG1655 RARE cultures

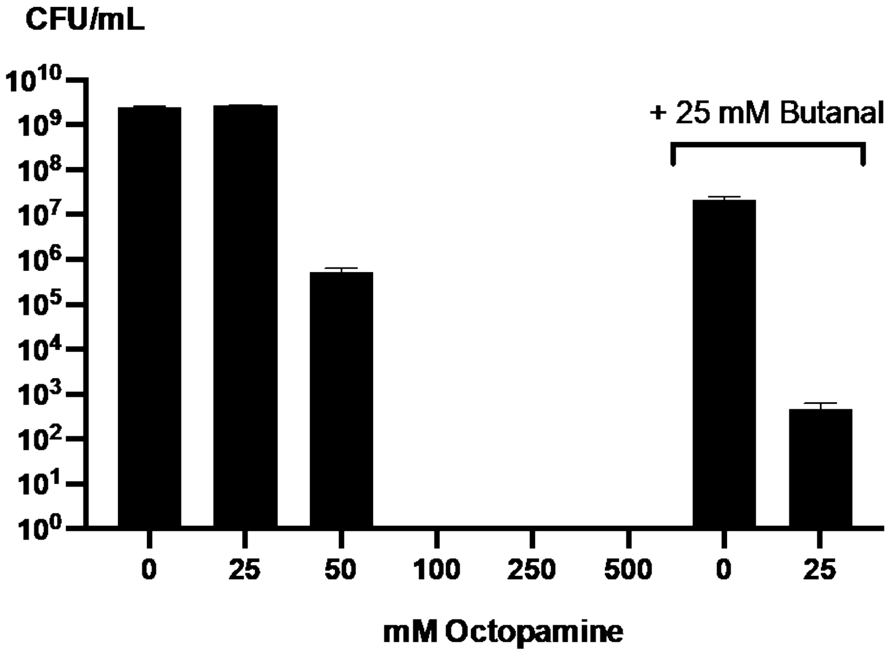

Figure S11: Comparing the effects of reaction components (octopamine (0-500 mM) ± butanal (25 mM)) at pH = 9 on the viability of *E. coli* MG1655 RARE

S9 Synthesis

S9.1 Synthesis of *N*-Methyltyramine isomers

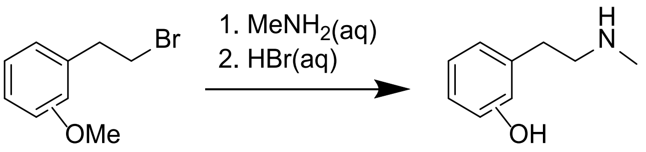

[2-(2-methoxyphenyl)ethyl](methyl)amine hydrobromide

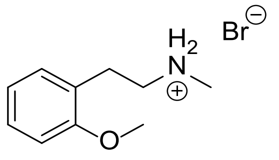

To a solution of methylamine (8.0 mL, 44 mmol, 40% in water) in THF (2 mL) at 20 °C was added a solution of 2-methoxypenylethyl bromide (1.0 g, 4.6 mmol) in THF (3.0 mL) dropwise over 20 min. The reaction mixture was stirred at 20 °C for 24 h before concentrating under reduced pressure. The residue was purified by flash chromatography (1−10% MeOH in DCM) to provide the title compound as a colourless solid (0.90 g, 78%). ^{1}H NMR (500 MHz, $CDCl_3$) δ 9.18 (s, 2H), 7.22 (td, *J* = 7.7, 0.7 Hz, 1H), 6.84 (dt, *J* = 7.5, 1.2 Hz, 1H), 6.82 – 6.75 (m, 2H), 3.78 (s, 3H), 3.23 (m, 4H), 2.72 (t, *J* = 5.6 Hz, 3H). ^{13}C NMR (126 MHz, $CDCl_3$) δ 160.1, 137.4, 130.1, 120.9, 114.4, 112.9, 55.3, 50.7, 33.1, 32.2.

[2-(3-methoxyphenyl)ethyl](methyl)amine hydrobromide

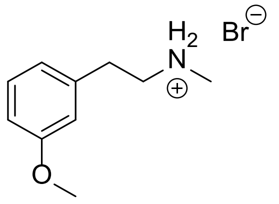

To a solution of methylamine (8.0 mL, 44 mmol, 40% in water) in THF (2 mL) at 20 °C was added a solution of 3-methoxypenylethyl bromide (1.0 g, 4.6 mmol) in THF (3.0 mL) dropwise over 20 min. The reaction mixture was stirred at 20 °C for 24 h before concentrating under reduced pressure. The residue was purified by flash chromatography (1−10% MeOH in DCM) to provide the title compound as a pale brown oil (0.99 g, 86%). ^{1}H NMR (500 MHz, $CDCl_3$) δ 9.04 (s, 2H), 7.28 – 7.23 (m, 2H), 6.91 (td, *J* = 7.4, 1.1 Hz, 1H), 6.87 (dd, *J* = 8.1, 1.1 Hz, 1H), 3.86 (s, 3H), 3.31 – 3.17 (m, 4H), 2.75 (t, *J* = 5.7 Hz, 3H). ^{13}C NMR (126 MHz, $CDCl_3$) δ 157.6, 130.9, 129.0, 124.4, 121.0, 110.6, 55.5, 49.1, 33.0, 27.7.

2-[2-(methylamino)ethyl]phenol

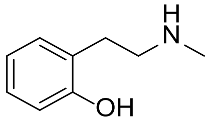

To a solution of [2-(2-methoxyphenyl)ethyl](methyl)amine hydrobromide (0.8 g, 3.3 mmol) in acetic acid (10 mL) was added aqueous HBr (47%, 10 mL) and the mixture was stirred at reflux for 6 h. The reaction mixture was concentrated under reduced pressure and the resulting residue basified to pH 8 with 1 M aqueous NaOH. The solution was washed with diethyl ether (2 x 30 mL). The aqueous fraction was then acidified to pH 2 with aqueous HCl (37%) and then made basic with aqueous ammonium hydroxide (28%). The solution was saturated with NaCl and then extracted with n-butanol (3 x 30 mL), dried over K_2CO_3 and concentrated to provide the title compound as a brown oil (0.12 g, 24%). ^{1}H NMR (500 MHz, MeOD) δ 7.06 – 6.97 (m, 2H), 6.79 – 6.71 (m, 1H), 6.68 (td, *J* = 7.4, 1.2 Hz, 1H), 2.85 – 2.80 (m, 4H), 2.42 (s, 3H). ^{13}C NMR (126 MHz, MeOD) δ 156.97, 145.60, 130.14, 127.20, 118.24, 115.95, 51.35, 34.10, 31.07.

3-[2-(methylamino)ethyl]phenol

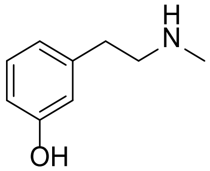

To a solution of [2-(3-methoxyphenyl)ethyl](methyl)amine hydrobromide (1.0 g, 4.1 mmol) in acetic acid (10 mL) was added aqueous HBr (47%, 10 mL) and the mixture was stirred at reflux for 3 h. The reaction mixture was concentrated under reduced pressure and the resulting residue basified (to pH 8) with 1 M aqueous NaOH. The solution was washed with diethyl ether (2 x 30 mL). The aqueous fraction was then acidified to pH 2 with aqueous HCl (37%) and then made basic with aqueous ammonium hydroxide (28%). The solution was saturated with NaCl and then extracted with n-butanol (3 x 30 mL), dried over K_2CO_3 and concentrated to provide the title compound as a brown oil (0.23 g, 37%). ^{1}H NMR (500 MHz, MeOD) δ 7.11 (t, *J* = 7.8 Hz, 1H), 6.71 – 6.67 (m, 1H), 6.67 – 6.62 (m, 2H), 2.87 – 2.61 (m, 4H), 2.40 (s, 3H). ^{13}C NMR (126 MHz, MeOD) δ 157.4, 140.9, 129.1, 119.4, 115.1, 112.9, 52.5, 35.0, 34.5.

S10 References

[1] S.N. Cohen, A.C.Y. Chang, L. Hsu, *PNAS.* **1972**, 69(8), 2110−2114

[2] H. Hashima, M. Hayashi, Y. Kamano et al. *Bioorg. Med. Chem*. **2000**, 8(7), 1757−1766

[3] S. F. Altschul, W. Gish, W. Miller, et al. *J .of Mol. Bio*. **1990** 215(3), 403−410
